# Biopsychosocial Mechanisms Linking Gender Minority Stress to HIV Comorbidities Among Black and Latina Transgender Women (LITE Plus): Protocol for a Mixed Methods Longitudinal Study

**DOI:** 10.2196/17076

**Published:** 2020-04-13

**Authors:** Ashleigh J Rich, Jennifer Williams, Mannat Malik, Andrea Wirtz, Sari Reisner, L Zachary DuBois, Robert Paul Juster, Catherine R Lesko, Nicole Davis, Keri N Althoff, Christopher Cannon, Kenneth Mayer, Ayana Elliott, Tonia Poteat

**Affiliations:** 1 School of Population & Public Health University of British Columbia Vancouver, BC Canada; 2 Department of Social Medicine University of North Carolina, Chapel Hill Chapel Hill, NC United States; 3 Bloomberg School of Public Health Johns Hopkins University Baltimore, MD United States; 4 Harvard Medical School Harvard University Boston, MA United States; 5 Department of Anthropology University of Oregon Eugene, OR United States; 6 Department of Psychiatry and Addiction University of Montreal Montreal, QC Canada; 7 Whitman Walker Health Washington, DC, DC United States; 8 TH Chan School of Public Health Harvard University Boston, MA United States; 9 National LGBT Health Education Center Boston, MA United States

**Keywords:** transgender persons, HIV, comorbidity, racial factors, stress, physiological, stress, biological

## Abstract

**Background:**

Black and Latina transgender women (TW) experience a disparate burden of HIV and related comorbidities, including poor mental health and cardiovascular disease (CVD) risks. Pervasive multilevel stigma and discrimination operate as psychosocial stressors for TW living with HIV and shape health disparities for this population. Gender-affirming hormone therapy (GAHT) is commonly used by TW to facilitate alignment of the body with gender identity; in the context of stigma, GAHT may both improve mental health and increase CVD risks.

**Objective:**

This study aims to quantify the longitudinal relationship between stigma and chronic stress among black and Latina TW living with HIV. Secondary objectives include identifying pathways linking chronic stress to HIV comorbidities and exploring chronic stress as a mediator in the pathway linking stigma and GAHT to CVD comorbidities.

**Methods:**

This US-based mixed methods longitudinal study will enroll a prospective cohort of 200 black and Latina TW living with HIV, collecting quantitative survey data, qualitative interviews, and biomarkers of chronic stress. Interviewer-administered surveys will include validated psychosocial measures of self-reported stigma and discrimination, perceived stress, CVD risk factors, mental health, access to gender-affirming care, coping, and social support. Medical record abstraction will collect data on GAHT use, CD4 count, HIV viral load, antiretroviral therapy, treatment, and comorbid conditions. Clinical measures will include physiological biomarkers as well as salivary and blood-based biomarkers of chronic stress. Survey data will be collected every 6 months (baseline, and 6, 12, 18, and 24 months), and biospecimens will be collected at baseline and at 12 and 24 months. A purposive subsample (stratified by use of GAHT and presence of depressive symptoms) of 20 to 30 TW living with HIV will be invited to participate in in-depth interviews at 6 and 18 months to explore experiences of intersectional stigma, chronic stress, and the role of GAHT in their lives.

**Results:**

This study was funded by the National Institute on Minority Health and Health Disparities in December 2018. The study community advisory board and scientific advisors provided critical input on study design. Recruitment began in October 2019 (n=29 participants as of submission) and data collection will continue through 2022, with publication of baseline results anticipated summer 2021.

**Conclusions:**

This study will focus on black and Latina TW living with HIV, an understudied health disparities population, advance both stigma and intersectionality research, and move chronic stress physiology research toward a more nuanced understanding of sex and gender. The comprehensive methodology will support the exploration of the role of exogenous estrogen in the pathways between stress and HIV comorbidities, elucidating the role of GAHT in the stress-health relationship. Finally, this study will provide longitudinal evidence of the impact of stigma-related chronic stress on the lives of black and Latina TW living with HIV integrating qualitative and quantitative data with psychosocial, clinical, and biological measures.

**International Registered Report Identifier (IRRID):**

DERR1-10.2196/17076

## Introduction

### HIV Disparities Among Transgender Women

Transgender women (TW) experience a disparate burden of HIV infection and HIV-related comorbidities [1]. A recent systematic review and meta-analysis of HIV among transgender populations in the United States found that HIV prevalence ranged from 14.2% by laboratory-confirmed diagnosis to 21.0% by self-report for TW [2]. Globally, overall HIV prevalence for TW is estimated to be 19%, 49-fold higher odds compared with that among cisgender adults [3]. Among TW, the heaviest burden of HIV is borne by black and Latina TW (BLTW) [4,5], with an overall HIV prevalence of 44.2% for black TW and 25.8% for Latina TW [2]. BLTW make up the majority of TW receiving HIV clinical care nationally in the United States [6,7].

### Multiple Pathways to Health Disparities for Transgender Women

Multilevel and multidimensional factors shape health disparities among TW living with HIV (TWLHIV). Individual (eg, sociodemographic and psychological), interpersonal (eg, violence, victimization, and gender-power dynamics), and structural (eg, stigma and discrimination) factors influence HIV-related outcomes among TWLHIV [4,8-11]. TWLHIV have high rates of living in poverty, facing housing insecurity, and lacking health insurance [7]. Given these barriers and experiences that fundamentally undermine health and well-being, TWLHIV are also less likely to adhere to antiretroviral therapy (ART) or to achieve durable HIV viral load suppression [7], thus facing elevated risk of mortality [12].

Due in part to stigma exposure, mental health and cardiovascular disease (CVD) disparities are common synergistic HIV comorbid conditions experienced by TWLHIV. In the largest national survey of transgender people to date (N=27,715), 40% reported ever attempting suicide and 39% reported psychological distress in the prior year, compared with 4.6% and 5% of the US general population, respectively [13]. A growing body of literature has identified associations between poor mental health and exposure to transgender stigma [14-17]. This may be exacerbated by HIV infection, as depression is the most common neuropsychiatric comorbidity among people living with HIV (PLWHIV), associated with poor adherence, lack of viral suppression, and increased CVD risk [18-20].

TW are also more likely to experience CVD risk factors, events, and mortality than cisgender adults (ie, nontransgender adults) [21,22]. In one of the largest studies published to date on TW and CVD, a retrospective mortality analysis of more than 1000 Dutch transgender adults, a 64% increased risk in CVD mortality was observed among TW compared with the general population [21]. Increased prevalence of CVD among PLWHIV has been attributed to chronic inflammation associated with HIV infection as well as higher prevalence of CVD risk factors (eg, obesity and diabetes) [22], risk behaviors (eg, smoking) [23,24], and stigma [25]. However, underlying psychologic and biologic pathways to CVD disparities among TWLHIV are poorly understood [26].

### Associations of Gender-Affirming Hormone Therapy With Chronic Stress and Cardiovascular Disease

Gender-affirming hormone therapy (GAHT), including exogenous estrogen, is commonly used by TW to facilitate alignment of the physical body with gender identity [13]. Approximately 75% to 95% of TW take GAHT at any point in time [27-29]. Access to GAHT is a community priority for TW [13] and can be a critical protective factor for HIV and other comorbidities [30], improving psychological functioning [21,31], facilitating care engagement [32], and improving ART adherence and viral suppression when provided in the context of HIV care [31,33-35]. In contrast to these benefits, GAHT has also been associated with an elevated CVD risk [21,22,27,36] and may potentiate CVD comorbidities among TWLHIV. Sex hormones play an important modulatory role in stress physiology [37,38]. They have been implicated in sex differences in CVD risk [39] and mental health [40]. However, previous studies in this area were conducted with cisgender people only or did not consider gender experience, limiting the ability to disaggregate hormonal effects from other gendered factors. Clinicians, scientists, and transgender communities have called for more longitudinal research on the effects of GAHT on health outcomes among TWLHIV [9,11], and especially among older TW who have been particularly understudied [36]. Specifically, data are needed on how GAHT may impact health disparity pathways for TWLHIV, who face both mental health and CVD comorbidities.

### Allostatic Load as a Biological Marker of Chronic Stress

Allostatic load (AL) refers to the cumulative *wear and tear* effects of chronic stress on the brain and body [41,42]. Models of AL demonstrate physiologic pathways linking psychosocial stressors to poorer physical and mental health [43]. AL derives from the concept of allostasis [44], the dynamic adaptive regulatory process of the body that seeks to maintain homeostasis during exposure to physical and psychological stressors [45]. As such, AL can be measured by assessing neuroendocrine, immune, metabolic, and cardiovascular biomarkers [45-47]. Dysregulation of these biomarkers has been linked to minority stress experiences, including stigma and discrimination, and is associated with poor mental health and CVD among racial and ethnic minorities [46-49], as well as elevated CVD risk among sexual minorities [50]. AL differs by sexual orientation [51]; however, studies examining pathways among transgender people who experience gender minority stress is limited [52,53], and there are few published studies of physiologic stress processes among PLWHIV [54]. Mechanisms linking chronic stress to comorbidities have not been elucidated for transgender people, particularly TW who experience stigma and discrimination on the basis of intersecting minority identities, such as race, gender identity, and HIV status (ie, intersectional stigma) [55,56]. Qualitative research among black women living with HIV has highlighted the inextricability of gender and race in understanding HIV-related stigma and its sequelae [57].

### Project Proposal

This novel prospective study aims to advance scientific knowledge of how intersectional stigma impacts HIV outcomes and comorbidities for black and Latina TWLHIV. Elucidating the multilevel pathways linking intersectional stigma to mental health and CVD comorbidities among TWLHIV, and exploring the role GAHT may play in mitigating or exacerbating these comorbidities in the context of gender minority stress, will advance scientific understanding of how stigma and discrimination become embodied. These advances in our understanding are key to our ability to identify ways clinical providers can more effectively tailor their care to meet the needs of this health disparity population. This project employs a mixed methods protocol in which biomarkers, clinical measures, survey responses, and in-depth interviews will be used to understand pathways shaping HIV comorbidities for black and Latina TWLHIV. Results will inform clinical practices and public health interventions that facilitate health care engagement and retention to promote health equity among black and Latina TWLHIV.

### Objectives

The primary objectives of this study are as follows:

1. To quantify the longitudinal relationship of stigma to chronic stress biomarkers in TWLHIV;
2. To identify pathways linking chronic stress biomarkers to comorbidities (ie, CVD and mental health) among TWLHIV; and
3. To explore chronic stress as a mediator in pathways linking stigma, GAHT, and HIV comorbidities.

## Methods

### Conceptual Model

**Figure 1:**
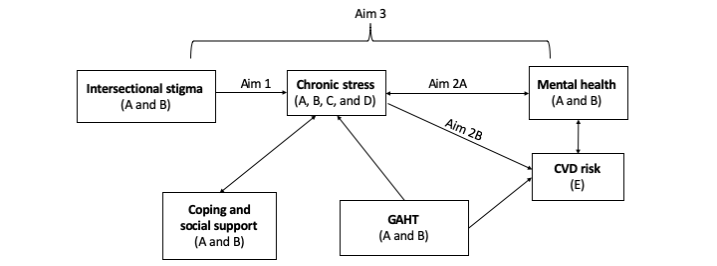
Hypothesized pathways linking stigma, stress, mental health, and cardiovascular disease outcomes among transgender women living with HIV to be measured using (A) survey, (B) qualitative interviews, (C) salivary cortisol, (D) allostatic load index, and (E) clinical measures. GAHT: gender-affirming hormone therapy; CVD: cardiovascular disease.

This study, named LITE Plus, draws on the Gender Minority Stress and Resilience Model (GMSR) [58,59] and chronic stress and AL constructs [60]. Our research uses an intersectionality [56] lens to recognize the multiple and intersecting levels of influence that shape health disparities among transgender populations. The GMSR posits that transgender mental and physical health disparities are driven by distal (eg, discrimination) and proximal (eg, internalized stigma) stressors that can be mitigated by factors such as social support (eg, community connectedness) [58]. AL models [61] allow the objective measurement of multisystem physiologic dysregulation caused by chronic stress [43] and have been applied to the study of a variety of health disparities [62]. An intersectionality approach centers the experience of people from multiply marginalized groups, such as black and Latina TWLHIV, and acknowledges that these multiple social identities are associated with disparate health outcomes via a confluence of social forces, such as racism, misogyny, transphobia, and HIV stigma [55]. In centering the experiences of historically marginalized groups, an intersectionality lens encourages the examination of oppressive social processes without the need for comparison with dominant groups [63]. We will examine biological pathways linking the chronic stress of intersectional stigma to mental health and CVD comorbidities among TWLHIV and the potential mitigating and exacerbating influences of social support and coping and GAHT along these pathways (Figure 1**)**.

### Study Design and Population

This study builds on the American Cohort to Study HIV Acquisition among Transgender Women in High Risk Areas (known as the LITE study) [64], which prospectively follows TW who are not living with HIV. Participants for the LITE Plus study will be recruited from two LITE clinical study sites (Fenway Health in Boston, MA, and Whitman-Walker Health in Washington, DC) that provide comprehensive health services to the lesbian, gay, bisexual, transgender, and queer communities, including HIV treatment and GAHT. Fenway consists of six satellite sites and Whitman-Walker consists of three health center sites. This mixed methods, multisite longitudinal study will enroll a prospective cohort of 200 black and Latina TWLHIV living in Boston, MA, and Washington, DC, areas (approximately 100 participants per site) and collect data every 6 months for 24 months total—at baseline and at 6, 12, 18, and 24 months. Interview-administered surveys will include validated psychosocial measures of stigma, perceived stress, CVD risk behaviors (eg, smoking), mental health, coping, and social support. Medical record abstraction will collect data on GAHT use, CD4 count, HIV viral load, ART medications, and comorbid conditions. Clinical measures will include height, weight, waist circumference, and blood pressure; salivary measures of cortisol; and blood samples for measures of physiological stress to be included in the AL index. Qualitative in-depth interviews will be conducted with a subset of 20 to 30 TWLHIV (up to 15 participants from each site, and based on participants’ use of GAHT and risk for depression) to explore their experiences of intersectional stigma, chronic stress, and the role of GAHT in their lives.

A community advisory board (CAB) has reviewed the study design and implementation plans to ensure that retention, recruitment, and data collection align with gender-affirming best practices. The LITE Plus CAB meets annually to receive study updates and give feedback on study progress. They are also consulted between meetings for feedback on study forms, methods, and surveys. The CAB will also review preliminary results and offer interpretations of findings as part of a community-engaged research model.

### Inclusion Criteria

Participants must have been assigned male sex at birth; identify as *female*, *woman*, *trans female*, *male-to-female*, or *woman of trans experience*; have a laboratory-confirmed HIV diagnosis; identify as black, Latina, and/or multiracial (inclusive of black and/or Latina identities); and be 18 years of age or older. All study participants must live in or near Boston, MA, or Washington, DC, speak English and/or Spanish, be mentally sound and capable of consenting and provide written consent to participate. To be eligible for home collection of salivary cortisol, participants must not be currently using any medicines containing steroids regardless of route of administration, have consistent access to a freezer for up to 4 weeks at a time, live within established geographic boundaries based on participant zip codes to facilitate staff retrieval of samples, and report willingness to collect three sets of six saliva samples over the course of the study. Eligibility for the qualitative interviews will be based on the participant history of GAHT and depression scores at baseline study visit.

### Study Procedures

#### Recruitment

Multiple strategies will be used to identify, recruit, and retain participants. Participants may be identified as eligible via their electronic medical records and invited to participate by study staff. Participants may also be recruited from the LITE study, when (1) they are excluded from the cohort at baseline because of a positive HIV test result, or (2) when they seroconvert during the follow-up, which ends their LITE cohort participation. Should we not reach target enrollment through clinic-based recruitment and recruitment through LITE, we will engage in community-based recruitment, in partnership with LITE. Community-based strategies will include venue-based recruitment from the gender-affirming community events and organizations frequented by TW and social media outreach.

#### Visit Schedule

**Figure 2:**
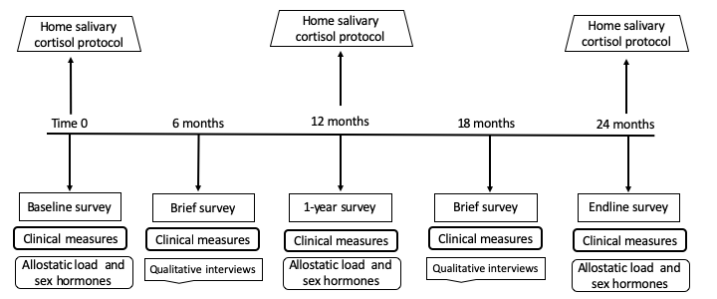
Overview of study visits and data collection intervals.

Surveys, clinical measures, and medical record abstraction will be collected at all study visits (every 6 months) over the 24-month study period. Phlebotomy and salivary cortisol self-collection training will occur at the baseline, 12-month, and 24-month visits. At the 6- and 18-month visits, a subgroup of participants will be invited to participate in in-depth interviews. The data collection schedule is detailed in Figure 2.

### Survey

Each participant will complete an interviewer-administered survey at all study visits over the 24-month study period. Participants will complete a longer survey at the baseline, 12-month, and 24-month visits, with brief surveys at the 6- and 18-month visits. Surveys will be administered by a trained interviewer via a tablet or desktop computer and will include validated measures used in prior research with transgender communities, where available. Survey domains include sociodemographics, validated psychosocial measures of stigma and discrimination, perceived stress, CVD risk behaviors, mental health, resilient coping, material social support, community connectedness, interpersonal violence, engagement in HIV care, and self-reported GAHT use. Baseline survey measures are described in Table 1.

**Table 1:** Key quantitative measures of the baseline survey.

Construct	Measures
ART^a^ adherence	Self-reported use, treatment interruptions, recent missed doses and reasons for missed doses [65], and challenges obtaining ART
Chronic stress	Perceived stress [66]
Coping and resilience	Brief resilient coping [67], gender identity pride [58], and community connectedness [58]
GAHT^b^	Duration, source, adherence, mode of delivery, frequency of monitoring of hormone levels in blood, discussion with health care provider regarding potential side effects, and perceptions of GAHT and ART interaction
Gender-affirming surgery	History of and need for
Health care access	Health insurance, health care access barriers, and typical health care setting
General health	History of diabetes, hypertension, high cholesterol, heart disease, blood clots, stroke, kidney disease, liver disease, cancer, obesity, etc, and perceived general health and healthy days (HRQOL-4^c^) [68]
HIV outcomes	Last time viral load measured, if applicable: reasons for not having viral load measured recently (within 6 months), suppressed viral load at last measurement
Intersectional stigma and discrimination	Fear of deportation [69,70], internalized HIV stigma (within social relationships) [71], internalized anticipated discrimination [72], everyday discrimination [73], internalized transphobia [58], and gender-related rejection [58]
Legal gender transition	Congruence between gender and preferred name and gender marker and name listed on IDs and records, and importance of congruent IDs and records [74]
Medical distrust	Trust in HIV care providers [75]
Mental health	Posttraumatic stress disorder (PCL-C^d^) [76], depressive symptomology (CESD-10^e^) [77], and history of suicidality and attempted suicide
STI^f^	History of STI testing and diagnosis, and history of hepatitis C
Sex work	Lifetime and recent history of engagement in sex work
Smoking history	Current smoking status [78] and pack-year smoking history [79]
Social support	Material social support [80]
Sociodemographics	Gender identity, sexual orientation, completed education, employment status, housing status and homelessness, immigration status, and material hardship [81]
Soft-tissue fillers	Lifetime use, location (ie, body parts), source of injections (eg, medical provider and parties)
Substance use	Past-year alcohol use (AUDIT-C^g^) [82] and past-year drug use (DAST-10^h^) [83]
Violence experiences	Lifetime and recent psychological, physical, and sexual violence (RCTS-2^i^) [84]

^a^ART: antiretroviral therapy.

^b^GAHT: gender-affirming hormone therapy.

^c^HROOL-4: Healthy Days Core Module health related quality of life measure.

^d^PCL-C: Post-traumatic stress disorder checklist- civilian version.

^e^CESD-10: Centre for Epidemiological Studies Depression Scale.

^f^STI: sexually transmitted infection.

^g^AUDIT-C: Alcohol Use Disorders Identification Test-Alcohol Consumption Questions.

^h^DAST-10: Drug Abuse Screening Test.

^i^RCTS-2: Revised Conflict Tactics Scales.

### Clinical Measures and Biospecimens

Clinical measures will be collected at each study visit, specifically height (at baseline only), weight, waist circumference, and blood pressure. Biospecimens (blood and saliva) will be collected at baseline and at 12- and 24-month follow-up visits to test for chronic stress biomarkers and CVD risk. At-home saliva collection will occur following the baseline, 12- and 24-month study visits. We chose to use saliva to test for stress-related biomarkers because at-home collection of saliva is considered minimally invasive [85]. Home salivary collection has proven feasible and effective in previous studies of stress and health among transgender men [37]. Furthermore, prior studies of at-risk populations (eg, abused women and low-income populations) have successfully utilized salivary analytes to study stress [86,87]. Salivary cortisol has been used extensively as a biomarker of stress in research settings, especially in studies examining psychological stress with repeated measurements [88], and is a useful component of the Allostatic Load Index (ALI) [89]. As cortisol follows a diurnal pattern throughout the day (peaking within 30 min of waking and declining over the course of the day), each participant will have a unique diurnal curve measured as awakening, 30-min postwake, and bedtime cortisol values [85,90,91]. Participants will be trained in the *passive drool* method of saliva self-collection [90] during baseline and receive booster trainings at the 12- and 24-month study visits. They will be provided with supplies to self-collect their saliva at home on two consecutive weekdays as soon as possible after the study visit. Participants will be instructed to store home-collected saliva samples in their freezers until study staff collect the samples, typically within 4 weeks. Samples will be subsequently stored in a −80 freezer at the site until they can be mailed to the designated laboratory for analysis. Sites will ship frozen saliva samples in batches to the Institute for Interdisciplinary Salivary Bioscience Research at the University of California Irvine every 6 months for analysis and storage.

In addition, study staff will draw 44 mL (3 tablespoons) of blood from each participant to test for estradiol, testosterone, progesterone, dehydroepiandrosterone-sulphate (DHEAS), total cholesterol, high-density lipoprotein (HDL), low-density lipoprotein, triglycerides, interleukin-6 (IL-6), tumor necrosis factor-alpha (TNF-alpha), C-reactive protein (CRP), fibrinogen, insulin, glycosylated hemoglobin (Hb), albumin, and creatinine levels. Blood samples will be sent to the LabCorp location nearest to the participant’s study site for analysis and results, once available, will be entered into the study database and shared with the participant (along with cortisol results) at their subsequent follow-up visits.

### Concomitant Medications

Study staff will collect information on concomitant medication use from participants, supplemented by medical record review. This will include all medications taken within 30 days of study visits. Concomitant medications of interest include ART, GAHT, and medications for blood pressure, diabetes, and heart disease.

### Medical Record Review

Medical record review and data extraction will be done for each participant within 1 week of each study visit. Participants will sign a Health Insurance Portability and Accountability Act (HIPAA) waiver as part of the study consent form. Participants that do not have medical records at Fenway or Whitman-Walker will sign a medical record request form that will be sent to their medical care provider. The medical record review and data extraction will be completed by trained data collectors using a standardized form. Specific information to be extracted from the medical records includes most recent CD4 count and HIV-RNA (viral load), current problem list and/or diagnosis codes, current medication list, and full history of all antiretroviral medications ever taken by the participant.

### In-Depth Interviews

At 6 and 18 months, a stratified purposive subsample of 20 to 30 participants (up to 15 at each site) will be invited to participate in qualitative interviews. Participants will be stratified based on self-reported baseline GAHT use and dichotomized depression scores. Stratification by these parameters will allow for qualitative exploration of relationships between GAHT use and mental health, consistent with the third study aim to assess relationships among GAHT, chronic stress, and HIV comorbidities. Participants in the qualitative data collection will take part in two in-depth life history calendar (LHC) interviews, conducted virtually with study staff via HIPAA-compliant audiovisual communication software. LHCs are a form of participant-empowered data collection that allows participants to take an active role in the data collection process and give feedback on how data are documented while providing a descriptive timeline of life experiences [92]. LHCs have been successfully adapted for electronic use, including among TW [93], allowing participants and interviewers to complete calendars in a virtual format [94]. Using virtual (computer-based) LHC [95], TWLHIV will work with interviewers to create a longitudinal timeline of their gender journeys, mental health, experiences of stigma and minority stress and use of GAHT, anchored by significant life course milestones. These interviews will be conducted by data collectors with specialized training in qualitative data collection. Participants may use their personal computers or computers made available to them at the study site.

### Incentives and Retention

Stepped incentives will be used to promote retention; participants must complete all study visit tasks to receive the incentive. Participants will be paid up to US $300 over the course of the study if they complete all study visit activities. Eligible participants may receive an additional US $150 for completing all of the home salivary collection and an additional US $60 for completing two in-depth interviews. An end-of-study bonus will be available for participants who are retained for 24 months and complete all study visits, making the incentive for the final visit US $80 for those participants. The detailed incentive structure is shown in Table 2.

The study clinical management system utilizes mobile phone text messaging and email to contact participants. The system automatically reminds participants of upcoming appointments and overdue visits, and sends other critical notifications regarding home salivary sample collection, distribution of incentives, etc. These notifications will serve as a first-line retention tool in combination with personalized messages and phone calls to encourage retention. Every effort will be made to coordinate study visits with routine clinical care visits for participants who also receive care at the study site.

All study information will be deidentified through the use of a unique identifier, which is generated for each participant at enrollment. Access to data by study staff will be on a role-based standard. All study staff will be trained in security and confidentiality procedures and will sign a confidentiality agreement before receiving access to any participant data. We will minimize the indirect disclosure of HIV status by referring to the study as a *health study for transgender women*.

**Table 2:** Stepped incentive structure.

Visit		Incentive amount (US $)	
Baseline		50	
6 months		50	
12 months		60	
18 months		60	
24 months		70	
**If eligible**			
	Baseline saliva collection		50
	12-month saliva collection		50
	24-month saliva collection		50
	In-depth Interview		30
	Study completion bonus		10

### Data Management and Tracking Participant Progress

LITE Plus uses the Clinical Trials Management System (CTMS) to track participant progress and automate study reminders. The automated features of the CTMS make it easy to remind participants of upcoming appointments and alert study staff when participant visit windows are closing. This HIPAA-compliant system provides a secure database in which to collect and store study data using the Transport Layer Security 2048-bit encryption. The CTMS is hosted by the Johns Hopkins University and has been customized to fit the LITE Plus protocol and workflow. Access to the CTMS is restricted to trained, certified data collectors and study team personnel.

### Quality Assurance and Control

Data collectors have been trained in study-specific quality assurance guidelines that include checks for data quality and completion during and after participant visits. Data collectors are trained to check study forms for completion before marking forms as *completed* in the CTMS. A quality assurance and control tracker has been developed for each site. Trained data collectors use this tracker to ensure that study forms are complete and consent forms are signed and dated.

### Statistical Methods and Analysis

#### Sample Size Calculation

Previous experience of this study team recruiting longitudinal cohorts of TWLHIV suggest 80% to 90% retention can be expected over the course of the study (ie, at the end of year 2, expected n=160) [96]. With a baseline sample of 200 TWLHIV, the power analysis shows a minimum detectable R^2^ of 4.3%, which is between Cohen thresholds of 2% and 13% for small and medium minimum detectable effect sizes, respectively, assuming N=200 followed by 20% attrition to yield a minimum analysis N of 160 for the analyses proposed for aims 1 and 2 [97,98]. A sample size of 160 achieves 80% power to detect an R^2^ of 0.043 attributed to one independent variable(s) using an *F* test with a significance level (alpha) of .050. The variables tested are adjusted for an additional 10 independent variable(s) with an R^2^ of 0.090.

#### Quantitative Analysis

Univariate and bivariate analyses and multivariable regression models will be used to compare levels and sources of stress between different levels of reported intersectional stigma, accounting for individual differences (eg, age, CD4 count, and HIV-RNA). Multivariable analyses will be used to measure the effects of the independent variables (eg, intersectional stigma) on dependent variables (eg, AL) while holding constant some factors (eg, age). For longitudinal analyses, marginal structural models will be used to account for repeated measures with time-dependent confounding and the potential for confounders at one point to be mediators at another [99].

#### Qualitative Analysis

LHC interviews will be transcribed verbatim. Spanish language transcripts will then be translated. All transcripts and LHC visual data will be uploaded into Atlas.ti Scientific Software Development GmbH to facilitate analysis. Documents will be coded using a priori codes created based on the interview guides. Codebooks will be modified iteratively based on emergent themes as coding progresses. Two coders will analyze the same transcripts separately. Any discrepancies will be discussed and resolved by peer debriefing and consensus with the PI, making final decisions should consensus not occur. The life course framework will be used to analyze codes for patterns and themes [100]. Member checking will be conducted with CAB members to enhance rigor of analyses.

## Results

### Recruitment to Date

The patient population at Whitman-Walker Health includes 1243 TW, of whom 235 are living with HIV. Of these TWLHIV, 71.0% (167/235) are black and 22.1% (52/235) are Latina. Based on a prior experience recruiting 112 black and Latina TWLHIV in the span of 3 months for a previous TW study at this site, the study team expects to recruit 100 TWLHIV from Whitman-Walker Health for this study without difficulty. Fenway’s patient population includes over 3500 transgender individuals of whom 1575 are TW. Among TWLHIV at Fenway, more than half are black and one-quarter are Latina. In 2017, up to 40 new transgender patients initiated care every month at Fenway, and the number of TWLHIV has steadily increased. In addition to existing patients, LITE Plus will also recruit potentially eligible TW from the LITE cohort who have been excluded at baseline for a positive HIV test result (n=65 to date) or who may seroconvert during the follow-up. If necessary, additional participants will be recruited using community-based strategies.

This study was funded in December 2018 by the National Institute on Minority Health and Health Disparities for a start date of December 1, 2018, and an end date of March 31, 2023. The study protocol was approved using a single institutional review at the University of North Carolina at Chapel Hill Institutional Review Board (IRB 18-2632). Recruitment began in October 2019, and we aim to enroll the full cohort (N=200) within 9 months of beginning the enrollment. Retention is expected to be 90% over the course of the first 12 months of the follow-up and at least 80% over the study duration (24 months). As of submission, 29 participants have been enrolled in the study. Publication of baseline results is expected in summer 2021.

### Main Study Constructs

Collected data will be used to construct the main study outcomes and predictors mentioned below.

#### Allostatic Load Index

As a way to examine cumulative stress effects on multiple body systems, AL is most often measured using an index that includes a battery of stress-related biomarkers with subclinical thresholds to quantify physiological dysregulations [101]. Over 100 studies have used a variety of AL algorithms that summarize neuroendocrine, immune, metabolic, and cardiovascular functioning [102] and predict disease better than existing approaches with single biomarkers [103,104]. Using 17 biomarkers (salivary cortisol, IL-6, TNF-alpha, DHEAS, insulin, glycosylated Hb, fibrinogen, CRP, total cholesterol, HDL, triglycerides, albumin, creatinine, systolic blood pressure, diastolic blood pressure, and BMI), we will calculate an ALI for each participant using an established, count-based approach that sums the number of dysregulated biomarkers using high-risk cutoffs based on the sample’s distribution of values for each biomarker, that is, the 75th percentile for biomarkers for which high levels are harmful, or the 25th percentile for which low levels are harmful [102]. A review of nearly 60 empirical studies suggests that ALIs incorporating similar subclinical ranges for numerous biomarkers (mean=10, range=4-17) predict clinical outcomes better than methods that address only clinical thresholds [102]. As each biomarker is dichotomized as 0 or 1, each has an equal weight in calculating the ALI [102] and ALI scores can range from 0 to 17 for this study. Based on an early review, the biomarkers included in this approach represent those most commonly used in the ALI literature [102]. ALI will be calculated for each participant using clinical and laboratory measures collected at baseline, 12 months, and 24 months to allow for observation of change over time.

#### Gender-Affirming Hormone Use and Cardiovascular Disease Risk

Cumulative GAHT exposure will be estimated as self-reported total number of years taking GAHT at baseline. For example, a participant who is 40 years old and started taking GAHT at 20 years old without interruption will be categorized as having 20 years of GAHT exposure. The Atherosclerotic CVD Pooled Cohort Equations (PCE) estimator [105] will be used to calculate CVD risk. As scores on the PCE are calibrated by sex, we will use a threshold of 1 year of current, continuous GAHT exposure at baseline as the cutoff for using the female-calibrated PCE, that is, participants with 1 year of GAHT will be classified as female for the PCE while those with less than 1 year of GAHT will use the male-calibrated PCE. We will assess the relationship between baseline ALI scores and changes in the PCE scores during the study follow-up period. PCE scores at 24 months will be used for the CVD risk outcome. Baseline GAHT exposure will be used to calibrate the PCE in terms of sex, to ensure any changes in the PCE risk over the study period are because of changes in the other biomarkers used to calculate the PCE.

#### Mental Health Comorbidity

Participants will complete brief validated mental health and substance use screeners during study visits, which have been implemented in previous research with transgender people [106]. The PCL-C Checklist-Civilian [76], a continuous measure of posttraumatic stress disorder symptom severity, and the Centre for Epidemiological Studies Depression Scale [77], a general population measure of depressive symptomology, will be used to assess recent mental health. Recent substance use will be assessed using the Alcohol Use Disorders Identification Test-Alcohol Consumption Questions [82], a brief screening tool for identifying individuals with an active alcohol use disorder, and the Drug Abuse Screening Test [83].

## Discussion

### Study Importance

This study is designed to address gaps in knowledge about the mechanisms of HIV comorbidities among PLWHIV from health disparity populations, specifically black and Latina TWLHIV. Our transdisciplinary, multiracial team of cisgender and transgender researchers are committed to authentic, empowering engagement with transgender communities such that research is conducted with, not on, communities [107]. Leveraging the infrastructure of an existing National Institutes of Health (NIH)-funded cohort study, LITE Plus will advance scientific knowledge by applying tools from the emerging field of psychoneuroimmunology to understand how intersectional stigma and discrimination become embodied as heath inequities [108]. LITE Plus will also clarify the role of sex hormones in stress physiology by examining how GAHT may both diminish psychological distress via gender affirmation and potentially increase CVD risk because of physiologic effects of estrogen.

Despite the heavy impact of HIV on TW and the confluence of comorbidity risk factors, remarkably little research has focused on TWLHIV [109]. This study will be among the first to address HIV comorbid conditions in this population and one of the few to prospectively assess the role of chronic stress biomarkers among PLWHIV [54,110]. In so doing, this study will advance both stigma and intersectionality research and move chronic stress physiology research toward a more nuanced understanding of sex and gender.

In filling the gap in research on GAHT among TWLHIV, this study will provide data to inform the likely complex relationship between the benefits and risks associated with estrogen in a population with an elevated risk of poor mental health and CVD related to HIV disease. Elucidating the role of sex hormones in the stress-health relationship may have implications for research with broader populations, including cisgender women. Finally, most existing studies of chronic stress, AL, and health have used cross-sectional designs [62]. Following study participants over the course of 24 months with both psychosocial and physiologic measures will strengthen the ability to make causal inferences about the nature of stress-health relationships in PLWHIV and the impact of coping and social support on health outcomes for TWLHIV, providing key data for future interventions.

### Limitations, Challenges, and Solutions

#### Sample Size and Attrition

Budget caps limit the sample size and, therefore, statistical power, especially if attrition is significant. Retaining participants is a challenge for all longitudinal studies, and differential loss to follow-up is a common threat to internal validity in prospective research. We have developed monthly enrollment targets and extensive protocols to support retention. If we cannot reach our target sample size at current sites, the Johns Hopkins Center for Transgender Health will support additional recruitment, and we will seek supplemental funding to include additional sites from the LITE study which has locations in Atlanta, Miami, and New York as well as in Boston and the Baltimore/DC area. We are also cognizant of potential recruitment fatigue on the part of potential TW participants at these health center sites, who have likely participated in other research studies, particularly given the relatively small numbers of TW in the patient population at each site [111].

#### Missing Data

Home salivary collection (as well as other data collection methods) are vulnerable to missing data, despite intensive tracking protocols. Missing data reduce power and may produce biased estimates. In addition to traditional statistical approaches to handling missing data [112], we will explore growth curve modeling as an emerging method for addressing missing data in salivary cortisol research [113].

#### Limited Observation Period

Cumulative stress exacts a toll over time; thus 24 months may not be enough to see a change. The existing literature from the MacArthur Aging study found significant associations between AL and health over approximately 2 years, providing support for the feasibility of our 24-month study design [101].

#### Cardiovascular Disease Risk Assessment

Cardiac events are likely to be rare over 24 months, and budget limitations do not allow for the use of more expensive assessments such as estimation of coronary artery calcium scores. However, the CVD risk measure used for this study is common in clinical practice and is likely to have real-world applicability [114]. In addition, the absence of an existing CVD risk measure validated for use among TWLHIV is a further limitation of this study. However, the adaptation of the PCE based on GAHT exposure in this longitudinal study will make an important contribution to our understanding of CVD risk for TWLHIV. We will also conduct sensitivity analyses assessing the impact of calibrating the PCE based on sex assigned at birth, gender identity, and GAHT exposure over the study period.

#### Nonprescribed Hormone Use

Nonprescription GAHT use is common and may be underreported. In addition to collecting self-report and medical record data, we will conduct laboratory tests for estradiol, testosterone, and progesterone. TWLHIV taking GAHT may differ from TWLHIV not taking GAHT in ways that confound the relationship between GAHT and CVD, for example, they may have stopped GAHT because of a diagnosis of CVD. Therefore, our assessment of self-reported GAHT exposure, along with laboratory tests for sex hormones and medical record extraction of CVD-related diagnoses and medications, will allow for pragmatic assessments that are applicable to clinical practice.

#### Unmeasured Confounders

The proposed study involves complex, overlapping systems with multiple interacting levels and domains. In such a system, it is impossible to know and control for all potential confounders. Although unmeasured confounders may threaten external validity, the findings that result from studies that address real-world complexity (eg, by enrolling people with multimorbidity) may provide findings that are most actionable in the real world of clinical care and social policy.

## Appendix

Multimedia Appendix 1 National Institute on Minority Health and Health Disparities funded grant reviews.

## Abbreviations

AL: allostatic load
ALI: allostatic load index
ART: antiretroviral therapy
BLTW: black and Latina transgender women
CAB: community advisory board
CRP: C-reactive protein
CTMS: Clinical Trials Management System
CVD: cardiovascular disease
DHEAS: dehydroepiandrosterone-sulphate
GAHT: gender-affirming hormone therapy
GMSR: Gender Minority Stress and Resilience Model
Hb: hemoglobin
HDL: high-density lipoprotein
HIPAA: Health Insurance Portability and Accountability Act
IL-6: interleukin-6
LHC: life history calendar
LITE: American Cohort to Study HIV Acquisition among Transgender Women in High Risk Areas
NIH: National Institutes of Health
PCE: pooled cohort equations
PLWHIV: people living with HIV
TNF-alpha: tumor necrosis factor-alpha
TW: transgender women
TWLHIV: transgender women living with HIV
