# Supplementary material for: Biopsychosocial Mechanisms Linking Gender Minority Stress to HIV Comorbidities Among Black and Latina Transgender Women (LITE Plus): Protocol for a Mixed Methods Longitudinal Study
Source: JMIR Res Protoc. 2020 Apr 13;9(4):e17076. doi: 10.2196/17076 (PMC7186865; doi:10.2196/17076)
Supplement: Multimedia Appendix 1 [file resprot_v9i4e17076_app1.pdf]

SUMMARY STATEMENT

(Privileged Communication)

PROGRAM CONTACT:
Rina Das
301-402-1366
dasr2@mail.nih.gov

Release Date: 06/15/2018
Revised Date: 06/15/2018

Application Number: 1 R01 MD013498-01

Principal Investigator

POTEAT, TONIA C.

Applicant Organization: JOHNS HOPKINS UNIVERSITY

Review Group: ZMD1 XLN (O1)
National Institute on Minority Health and Health Disparities Special Emphasis Panel
Mechanisms of Disparities for HIV- Related Co-morbidities in Health Disparity Populations
AIDS - EXP. REV.

Meeting Date: 05/30/2018
Council: AUG 2018
Requested Start: 09/01/2018
RFA/PA: MD18-002
PCC: IBB01RD
Dual IC(s): DE

Project Title: Biopsychosocial Mechanisms Linking Gender Minority Stress to HIV Comorbidities

SRG Action: Impact Score:33
Next Steps: Visit https://grants.nih.gov/grants/next_steps.htm
Human Subjects: 30-Human subjects involved - Certified, no SRG concerns
Animal Subjects: 10-No live vertebrate animals involved for competing appl.
Gender: 2A-Only women, scientifically acceptable
Minority: 2A-Only minorities, scientifically acceptable
Children: 3A-No children included, scientifically acceptable

Project Year	Direct Costs Requested	Estimated Total Cost
1	418,571	650,616
2	448,588	697,273
3	449,513	698,711
4	449,947	699,386
5	431,452	670,637
TOTAL	2,198,071	3,416,623

ADMINISTRATIVE BUDGET NOTE: The budget shown is the requested budget and has not been adjusted to reflect any recommendations made by reviewers. If an award is planned, the costs will be calculated by Institute grants management staff based on the recommendations outlined below in the COMMITTEE BUDGET RECOMMENDATIONS section.
EARLY STAGE INVESTIGATOR
NEW INVESTIGATOR

1R01MD013498-01 POTEAT, TONIA

EARLY STAGE INVESTIGATOR
NEW INVESTIGATOR

RESUME AND SUMMARY OF DISCUSSION: This application is submitted in response to RFA-MD18-002, Mechanisms of Disparities for HIV-Related Co-morbidities in Health Disparity Populations (R01-Clinical Trial Not Allowed). The purpose of this proposal is to investigate the pathways linking stigma and physiologic stress to HIV comorbidities among Black and Latina transgender women (TW). There is relatively little research on this population, so this study will fill a major gap, and the results could have a significant impact on advancing transgender research and reducing health disparities. The investigative team is very experienced, and the innovative approach will utilize longitudinal study, in-depth-interviews and quantitative psychosocial research with clinical and biological measures to identify mechanisms linking stigma with sex hormones and HIV comorbidities, as well as linking chronic stress biomarkers with HIV comorbidities. Promising preliminary data and prior related research is the basis for a sound premise of the proposed study. However, some concerns are raised about a lack of details in both feasibility and validity of the approach, and in the analytic components for quantitative data proposed in the study. Overall, this application is rated excellent with high impact.

DESCRIPTION (provided by applicant): Despite a high prevalence of HIV among gender minority women, very little is known about HIV comorbid conditions in this population. Understanding the mechanisms of underlying HIV-related comorbidities in this health disparity population is an important step toward advancing health equity. The address this gap in knowledge, the study seeks to elucidate pathways linking stigma and physiologic stress to HIV comorbidities among Black and Latina gender minority women. The specific aims of the project include to: (1) quantify the longitudinal relationship of stigma to chronic stress biomarkers; (2) identify pathways linking chronic stress biomarkers to HIV comorbidities; and (3) examine the role of chronic stress in pathways linking stigma, sex hormones, and HIV comorbidities. These aims will be met using existing NIH-funded cohort platforms to enroll and follow 200 participants in a 24-month, mixed-methods, prospective study to measure stigma, biomarkers of chronic stress, sex hormones, mental health, and CVD risk. Data will be collected at baseline, 6-, 12-, 18-, and 24-months. This longitudinal study blends in-depth-interviews and quantitative psychosocial research with clinical and biological measures to advance understanding of the complex relationships between social experiences and physical health. This innovative research will identify mechanisms underlying associations between stigma and HIV comorbidities. It has important implications for interventions with health disparity populations living with HIV and will provide novel information that can be used to guide clinical practice and reduce health disparities.

PUBLIC HEALTH RELEVANCE
A disproportionate number of gender minority women are living with HIV in the United States, yet information about mechanisms of disparities for their HIV-related comorbidities is quite limited. This research takes a step toward filling this gap by building on the existing infrastructure of current NIH-funded prospective cohorts. The goal of this study is to investigate the pathways linking stigma and physiologic stress to HIV comorbidities among Black and Latina gender minority women.

CRITIQUES: The critiques below were prepared by the reviewers assigned to this application. These commentaries do not necessarily reflect the position of the authors at the close of the group discussion, nor the final majority opinion of the group, although reviewers are asked to amend their critiques if their position changed during the discussion. The resume and summary of discussion, together with the sections at the end of the summary statement, which summarize the committee's final opinion on the use of human subjects, the inclusion of women, minorities, and children, the use of vertebrate animals, and budget are the authoritative representation of the final outcome of group discussion. If there is any discrepancy between the peer reviewers' commentaries and the numerical score on the face page of

this summary statement, the numerical score should be considered the most accurate representation of the final outcome of the group discussion.

CRITIQUE 1

Significance: 3
Investigator(s): 1
Innovation: 2
Approach: 8
Environment: 1

Overall Impact:

The proposed project aims to examine chronic stress mechanisms linking intersectional stigma to mental health and cardiovascular disease (CVD) comorbidities among transgender women (TW) and to explore the role of gender affirming hormone therapy (GAHT). As detailed in the well-laid out premise, there is a great need for the proposed work, given the levels of stress and stigma experienced, the burden of HIV and other comorbidities among TW populations and the prevalence of mental health and CVD, and the limited knowledge of GAHT. Aims are to: 1. Quantify the relation between stigma and chronic stress biomarkers among TW living with HIV (TWLHIV); 2. Identify pathways linking chronic stress biomarkers to comorbidities among TWLHIV; and 3. to explore chronic stress as a mediator in between stigma, GAHT and HIV comorbidities. This study is significant because it will further our understanding of chronic stress, both experienced and biologic, and comorbidities among this under studied group. Further, the ability to tease out the impact of GAHT is very significant. Despite the many strengths, the impact is weakened substantially by limitations to the scientific rigor / approach, particularly for the quantitative components. Many details are lacking in important measurement components and there are major flaws in the analytic approach. As written, the proposed study is expected to have a moderate impact.

1. Significance:

Strengths

- If aims are achieved, the project will provide a deeper understanding of health disparity pathways among TWLHIV—specifically, the role of chronic stress and stigma on mental health and CVD comorbidities, as well as the impact of GAHT.
- Potential to further the field in our understanding of chronic stress as a result of gender versus sex/hormonal effects, as well as the impact of intersectional stigma on chronic physiologic stress among PLWHIV as well as on comorbidities. Examination of the role of GAHT is particularly significant given the stress reducing aspects but potentially simultaneously increased CVD risk as a result of GAHT.

Weaknesses

- While focusing on TWLHIV is an important population, it seems like a missed opportunity to also include other gender minority groups.

2. Investigator(s):

Strengths

- Excellent interdisciplinary team, led by an early stage but accomplished investigator, with expertise needed to carry out the proposed work.

Weaknesses

- None noted.

3. Innovation:

Strengths

- Examination of the role of exogenous estrogen in the pathways between stress and comorbidities.
- Integration of qualitative data to further inform our understanding of stress and comorbidities and GAHT use in this population.
- Focuses on an understudied population –TWLHIV – and physiologic stress in an HIV population, which has also been understudied.

Weaknesses

- None noted.

4. Approach:

Strengths

- Utilization of both quantitative and qualitative data as well as a longitudinal design for quantitative analyses.
- Multiple indicators across biologic systems for allostatic load measurement.
- Feasibility of cohort recruitment given environment, existing studies and organizations involved.

Weaknesses

- There are a number of details missing that speak to both feasibility and validity of the approach. For example, how is TW status identified for enrollment? Are BP, BMI, WHR measured via the medical record or by study staff regularly? If the latter, the validity of that data is questionable. How is the CVD risk estimator administered and how is it operationalized? Is ART status to be defined as status in general or by type of ART, given that some may have more of an impact on metabolic indicators (e.g., insulin resistance and dyslipidemia)?
- Why 3 cortisol measures and not 4, particularly to get the cortisol awakening response (CAR)?
- How will intersectional stigma be operationalized or defined? That is a key exposure and there is no detail on how this will be defined or examined.
- There are major limitations to the analytic approach for quantitative data. First, none of the analyses will take into account repeated measures on each participant and potential biases that may result from that design effect given that responses are not independent. Second, the longitudinal modeling of potential confounders also presents an issue given that some confounders (e.g., coping, social support, smoking) may be confounders at one point but mediators at another. This should be discussed and analyzed (e.g., marginal structural models). Finally, there are also limitations to the Baron and Kenny method for mediation that should be addressed (e.g., Valeri, Linda, and Tyler J. VanderWeele. "Mediation analysis allowing for exposure–mediator interactions and causal interpretation: Theoretical assumptions and implementation with SAS and SPSS macros." *Psychological methods* 18.2 (2013): 137.).

5. Environment:

Strengths

- Excellent.

Weaknesses

- None noted.

Protections for Human Subjects:

Acceptable Risks and/or Adequate Protections

Inclusion of Women, Minorities and Children:

- Sex/Gender: Distribution justified scientifically
- Race/Ethnicity: Distribution justified scientifically
- For NIH-Defined Phase III trials, Plans for valid design and analysis:
- Inclusion/Exclusion of Children under 18: Excluding ages <18; justified scientifically

Vertebrate Animals:

Not Applicable (No Vertebrate Animals)

Applications from Foreign Organizations:

Not Applicable (No Foreign Organizations)

CRITIQUE 2

Significance: 2
Investigator(s): 1
Innovation: 2
Approach: 1
Environment: 1

Overall Impact:

This study will elucidate pathways linking stigma and physiologic stress to HIV comorbidities among Black and Latina gender minority women. The project will: (1) quantify the longitudinal relationship of stigma to chronic stress biomarkers; (2) identify pathways linking chronic stress biomarkers to HIV comorbidities; and (3) examine the role of chronic stress in pathways linking stigma, sex hormones, and HIV comorbidities. Existing NIH-funded cohort platforms will support enrollment of 200 participants in a 24-month, mixed-methods, prospective study to measure stigma, biomarkers of chronic stress, sex hormones, mental health, and CVD risk. Data will be collected at baseline, 6-, 12-, 18-, and 24-months. This research will identify mechanisms underlying associations between stigma and HIV comorbidities, and has implications for interventions with health disparity populations living with HIV. Significance is high, the research team is outstanding with highly relevant experience and expertise, innovation is strong, and approach is well considered in that potential design weakness are acknowledged with reasonable plans for addressing concerns. Overall, this is an outstanding/exceptional proposal.

1. Significance:

Strengths

- As noted by the investigators, "…transgender women (TW) are an NIMHD-designated disparities population who bear a disparate burden of HIV and related comorbidities. More than one in five (22%) TW are estimated to be living with HIV (TWLHIV), and 80% of TWLHIV are racial/ethnic minorities. …TWLHIV are more likely to live in poverty, have major depression, smoke cigarettes, and have CD4 <200. They are also less likely to adhere to antiretroviral medications or have durable viral suppression, thus face a 4-fold higher risk of death than virally suppressed individuals…"
- The investigators will systematically examine indices of chronic stress (biological and behavioral), contextualize stress within sociocultural and health environments, and will address implications for changes in the care environment.

Weaknesses

- None noted.

2. Investigator(s):

Strengths

- PI, Tonia Poteat, PhD, is an early career investigator with extensive experience with the population of interest, TWLHIV, and a strong and relevant publication record. The PI is supported by: Co-I Andrea Wirtz, PhD, MPH, an Assistant Scientist of Epidemiology, who has a strong and relevant publication record and a decade of relevant research experience as a research associate and project director (10% effort); Co-I Keri Altoff, Ph.D., Associate Professor of Epidemiology, and a member of the statistics in Epidemiology group. Dr. Altoff co-directs the NA-ACCORD study – a 20 site cohort study of HIV incidence among TW (2.5% effort); Co-I Todd Brown, MD MPH, Professor of Medicine in the Division of Endocrinology and Metabolism. He is a MAC co-investigator with extensive relevant experience (5% effort no salary); Co-I Wendy Post, MD MS, a cardiologist with extensive relevant experience with respect to CVDs (1% effort). The Directors of Whitman Walker and Fenway Health are both formally involved in the research effort (10% effort) and will each lead the site research teams. Scientific advisors/consultants also contribute expertise. An outstanding research team.

Weaknesses

- None noted.

3. Innovation:

Strengths

- Focus on, and access to, transgender women living with HIV.
- Focus on the impact of stigma-related chronic stress – operationally defined as allostatic load.
- Examining the role of exogenous estrogen on stress pathways and comorbid conditions with respect to mental health and cardiovascular disease (CVD) outcomes.
- Longitudinal study design to examine above.

Weaknesses

- While longitudinal/prospective, many events/factors influencing health may occur prior to the year under study – influencing disease progression without direct attribution.

4. Approach:

Strengths

- Strong ties to community partners over time – including Whitman Walker in Washington DC and Fenway Health in Boston, MA, with a well-documented history of access to and recruitment of TWLHIV.
- Strong preliminary data demonstrating feasibility of sampling and conduct of research, as well as disparities in mental health among TWLHIV compared to non-transgendered men and women.
- Gender Minority Stress and Resilience Model (GMSR), allostatic load models, and intersectionality are appropriate models to guide this research.
- Study design is very straightforward and well-articulated.
- Instruments and measures well described and implementation appears feasible.
- Data analysis plan is thorough and well considered and power analysis indicates adequate sample size
- Well described limitations and challenges – with reasonable actions to mitigate threats to integrity of findings.

Weaknesses

- None noted.

5. Environment:

Strengths

- Environments appropriate and supportive of proposed activities.

Weaknesses

- None noted.

Protections for Human Subjects:

Acceptable Risks and/or Adequate Protections

Data and Safety Monitoring Plan (Applicable for Clinical Trials Only):

Acceptable

Inclusion of Women, Minorities and Children:

- Sex/Gender: Distribution justified scientifically
- Race/Ethnicity: Distribution justified scientifically
- For NIH-Defined Phase III trials, Plans for valid design and analysis: Not applicable
- Inclusion/Exclusion of Children under 18: Excluding ages <18; justified scientifically

Vertebrate Animals:

Not Applicable (No Vertebrate Animals)

Biohazards:

Not Applicable (No Biohazards)

Applications from Foreign Organizations:

Not Applicable (No Foreign Organizations)

Select Agents:

Not Applicable (No Select Agents)

Resource Sharing Plans:

Acceptable

Authentication of Key Biological and/or Chemical Resources:

Acceptable

Budget and Period of Support:

Recommend as Requested

CRITIQUE 3

Significance: 2
Investigator(s): 1
Innovation: 2
Approach: 2
Environment: 1

Overall Impact:

This application proposes to investigate mechanisms linking stigma and physiologic stress of comorbidities among black and Latino Transgender women with HIV infection. It is estimated that over 20% of these women suffer from HIV-infection and over 80% of them belong to racial/ethnic minorities. Information on relationship of these stigma related challenges on the physiologic pathways is under estimated particularly among these Transgender minority women - a major proportion them also live in poverty. Such a study will elucidate our knowledge on the longitudinal relationship on stress biomarkers; link pathways of stigma with sex hormones and HIV comorbidities as well as linking chronic stress markers with HIV comorbidities. Since availability of such transgender population is limited, this application stands a very high probability to advance our knowledge in this field.

1. Significance:

Strengths

- Both mental health and CVD are linked to physiologic chronic stress. but such relationships have not been fully investigated among HIV infected transgender women.

- Transgender women frequently use gender affirming therapy which may also lead to CVD, but data are needed to establish this relationship and the involved mechanisms.
- Relationship between chronic stress as a mediator in pathways linking stigma, gender affirming hormone therapy and HIV comorbidities remain under investigated.
- The progress in this field may also be related to the availability of transgender women and the present application seems to have the requisite.

Weaknesses

- None noted.

2. Investigator(s):

Strengths

- Tonia Poteat is a new investigator but seems to have requisite experience and is being assisted by a number of qualified co-investigators.

Weaknesses

- There are 11 co-investigators or consultants, it is not clear how consultations with them will be carried out.

3. Innovation:

Strengths

- Investigations on HIV-infected transgender population are highly innovative since there requisite number at one place is not common and secondly since this population suffers from chronic stressors, and therefore, investigating relationships with biology particularly CVD is highly desired area of research.

Weaknesses

- None noted.

4. Approach:

Strengths

- Quantification of the longitudinal relationship of stigma to chronic stress markers is an outstanding objective.
- Linking pathways of chronic stress biomarkers to HIV related co-morbidities appears to be highly desirable.
- A study of pathways involved in examining to link stigma, sex hormones and HIV-comorbidities has not been fully examined in the study population which is itself not available in requisite numbers at all centers.

Weaknesses

- None noted.

5. Environment:

Strengths

- Outstanding.

Weaknesses

- None noted.

Protections for Human Subjects:

Acceptable Risks and/or Adequate Protections

Data and Safety Monitoring Plan (Applicable for Clinical Trials Only):

Acceptable

Inclusion of Women, Minorities and Children:

- Sex/Gender: Distribution justified scientifically
- Race/Ethnicity: Distribution justified scientifically
- For NIH-Defined Phase III trials, Plans for valid design and analysis: Not applicable
- Inclusion/Exclusion of Children under 18: Excluding ages <18; justified scientifically

Vertebrate Animals:

Not Applicable (No Vertebrate Animals)

THE FOLLOWING SECTIONS WERE PREPARED BY THE SCIENTIFIC REVIEW OFFICER TO SUMMARIZE THE OUTCOME OF DISCUSSIONS OF THE REVIEW COMMITTEE, OR REVIEWERS' WRITTEN CRITIQUES, ON THE FOLLOWING ISSUES:

PROTECTION OF HUMAN SUBJECTS: ACCEPTABLE

INCLUSION OF WOMEN PLAN: ACCEPTABLE

INCLUSION OF MINORITIES PLAN: ACCEPTABLE

INCLUSION OF CHILDREN PLAN: ACCEPTABLE

COMMITTEE BUDGET RECOMMENDATIONS: The budget was recommended as requested.

Footnotes for 1 R01 MD013498-01; PI Name: POTEAT, TONIA C.

NIH has modified its policy regarding the receipt of resubmissions (amended applications). See Guide Notice NOT-OD-14-074 at http://grants.nih.gov/grants/guide/notice-files/NOT-OD-14-074.html. The impact/priority score is calculated after discussion of an application by averaging the overall scores (1-9) given by all voting reviewers on the committee and multiplying by 10. The criterion scores are submitted prior to the meeting by the individual reviewers assigned to an application, and are not discussed specifically at the review meeting or calculated into the overall impact score. Some applications also receive a percentile ranking. For details on the review process, see http://grants.nih.gov/grants/peer_review_process.htm#scoring.

MEETING ROSTER

National Institute on Minority Health and Health Disparities Special Emphasis Panel
NATIONAL INSTITUTE ON MINORITY HEALTH AND HEALTH DISPARITIES
Mechanisms of Disparities for HIV- Related Co-morbidities in Health Disparity Populations

ZMD1 XLN (O1)
05/30/2018

Notice of NIH Policy to All Applicants: Meeting rosters are provided for information purposes only. Applicant investigators and institutional officials must not communicate directly with study section members about an application before or after the review. Failure to observe this policy will create a serious breach of integrity in the peer review process, and may lead to actions outlined in NOT-OD-14-073 at https://grants.nih.gov/grants/guide/notice-files/NOT-OD-14-073.html and NOT-OD-15-106 at https://grants.nih.gov/grants/guide/notice-files/NOT-OD-15-106.html, including removal of the application from immediate review.

CHAIRPERSON(S)

KUMAR, MAHENDRA, PHD
PROFESSOR AND CHIEF, PSYCHONEUROENDOCRINOLOGY AND PSYCHONEUROTRANSMITTERS LAB
DEPARTMENT OF PSYCHIATRY AND BEHAVIORAL SCIENCES
MEMBER, FACULTY SENATE & FACULTY COUNCIL
MILLER SCHOOL OF MEDICINE
UNIVERSITY OF MIAMI
MIAMI, FL 33136

MEMBERS

CAMPA, ADRIANA, PHD
CHAIR AND ASSOCIATE PROFESSOR
DEPARTMENT OF DIETETICS AND NUTRITION
ROBERT STEMPEL COLLEGE OF PUBLIC HEALTH AND SOCIAL WORK
FLORIDA INTERNATIONAL UNIVERSITY
MIAMI, FL 33199

IGUCHI, MARTIN YONEO, PHD
SENIOR BEHAVIORAL SCIENTIST
RAND CORPORATION
SANTA MONICA, CA 90407

KAPADIA, FARZANA, PHD, MPH
ASSOCIATE PROFESSOR OF GLOBAL PUBLIC HEALTH & POPULATION HEALTH
DEPARTMENT OF EPIDEMIOLOGY
COLLEGE OF GLOBAL PUBLIC HEALTH
NEW YORK UNIVERSITY
NEW YORK, NY 10003

QI, QIBIN, PHD
ASSISTANT PROFESSOR
ASSOCIATE DIRECTOR, CENTER FOR POPULATION COHORTS
DEPARTMENT OF EPIDEMIOLOGY & POPULATION HEALTH
ALBERT EINSTEIN COLLEGE OF MEDICINE
BRONX, NY 10461

ROSENBERG, ELI SAMUEL, PHD
ASSOCIATE PROFESSOR
DEPARTMENT OF EPIDEMIOLOGY AND BIOSTATISTICS
SCHOOL OF PUBLIC HEALTH
UNIVERSITY AT ALBANY, STATE UNIVERSITY OF NEW YORK
RENSSELAER, NY 12144

SCHLECHT, NICOLAS, PHD
PROFESSOR OF ONCOLOGY
DEPARTMENT OF CANCER PREVENTION & CONTROL
ROSWELL PARK COMPREHENSIVE CANCER CENTER
BUFFALO, NY 14263

THAMES, APRIL D, PHD
ASSOCIATE PROFESSOR
CLINICAL NEUROPSYCHOLOGIST
DEPARTMENT OF PSYCHOLOGY
UNIVERSITY OF SOUTHERN CALIFORNIA
LOS ANGELES, CA 90089

THEALL, KATHERINE P, PHD, MPH
ASSOCIATE PROFESSOR
DEPARTMENT OF GLOBAL COMMUNITY HEALTH AND BEHAVIOR SCIENCES
SCHOOL OF PUBLIC HEALTH AND TROPICAL MEDICINE
TULANE UNIVERSITY
NEW ORLEANS, LA 70112

WIGFALL, LISA TISDALE, PHD
ASSISTANT PROFESSOR
DEPARTMENT OF HEALTH AND KINESIOLOGY
TEXAS A&M UNIVERSITY
COLLEGE STATION, TX 77843

SCIENTIFIC REVIEW OFFICER

NAN, XINLI, MD, PHD
SCIENTIFIC REVIEW OFFICER
SCIENTIFIC REVIEW BRANCH
OFFICE OF EXTRAMURAL RESEARCH ADMINISTRATION
NATIONAL INSTITUTE ON MINORITY HEALTH AND HEALTH DISPARITIES, NATIONAL INSTITUTES OF HEALTH
BETHESDA, MD 20892

EXTRAMURAL SUPPORT ASSISTANT

DAVIS, CRYSTAL
SUPPORT ASSISTANT
SCIENTIFIC REVIEW BRANCH
OFFICE OF EXTRAMURAL RESEARCH ADMINISTRATION
NATIONAL INSTITUTE ON MINORITY HEALTH AND
HEALTH DISPARITIES, NATIONAL INSTITUTES OF HEALTH
BETHESDA, MD 20892

Consultants are required to absent themselves from the room during the review of any application if their presence would constitute or appear to constitute a conflict of interest.
